# DSC-PWI presurgical differentiation of grade 4 astrocytoma and glioblastoma in young adults: rCBV percentile analysis across enhancing and non-enhancing regions

**DOI:** 10.1007/s00234-024-03385-0

**Published:** 2024-06-05

**Authors:** Albert Pons-Escoda, Pablo Naval-Baudin, Mildred Viveros, Susanie Flores-Casaperalta, Ignacio Martinez-Zalacaín, Gerard Plans, Noemi Vidal, Monica Cos, Carles Majos

**Affiliations:** 1 Radiology Department, Hospital Universitari de Bellvitge, Barcelona, Spain; 2 Neuro-oncology Unit, Institut d’Investigació Biomèdica de Bellvitge- IDIBELL, Barcelona, Spain; 3 Facultat de Medicina i Ciències de La Salut, Universitat de Barcelona (UB), Barcelona, Spain; 4 Diagnostic Imaging and Nuclear Medicine Research Group, Institut d’Investigació Biomèdica de Bellvitge- IDIBELL, Barcelona, Spain; 5 Neurosurgery Department, Hospital Universitari de Bellvitge, Barcelona, Spain; 6 Pathology Department, Hospital Universitari de Bellvitge, Barcelona, Spain

**Keywords:** Brain neoplasms, Astrocytoma, Glioblastoma, Perfusion imaging, Magnetic resonance imaging

## Abstract

**Purpose:**

The presurgical discrimination of IDH-mutant astrocytoma grade 4 from IDH-wildtype glioblastoma is crucial for patient management, especially in younger adults, aiding in prognostic assessment, guiding molecular diagnostics and surgical planning, and identifying candidates for IDH-targeted trials. Despite its potential, the full capabilities of DSC-PWI remain underexplored. This research evaluates the differentiation ability of relative-cerebral-blood-volume (rCBV) percentile values for the enhancing and non-enhancing tumor regions compared to the more commonly used mean or maximum preselected rCBV values.

**Methods:**

This retrospective study, spanning 2016–2023, included patients under 55 years (age threshold based on World Health Organization recommendations) with grade 4 astrocytic tumors and known IDH status, who underwent presurgical MR with DSC-PWI. Enhancing and non-enhancing regions were 3D-segmented to calculate voxel-level rCBV, deriving mean, maximum, and percentile values. Statistical analyses were conducted using the Mann-Whitney U test and AUC-ROC.

**Results:**

The cohort consisted of 59 patients (mean age 46; 34 male): 11 astrocytoma-4 and 48 glioblastoma. While glioblastoma showed higher rCBV in enhancing regions, the differences were not significant. However, non-enhancing astrocytoma-4 regions displayed notably higher rCBV, particularly in lower percentiles. The 30th rCBV percentile for non-enhancing regions was 0.705 in astrocytoma-4, compared to 0.458 in glioblastoma (*p* = 0.001, AUC-ROC = 0.811), outperforming standard mean and maximum values.

**Conclusion:**

Employing an automated percentile-based approach for rCBV selection enhances differentiation capabilities, with non-enhancing regions providing more insightful data. Elevated rCBV in lower percentiles of non-enhancing astrocytoma-4 is the most distinguishable characteristic and may indicate lowly vascularized infiltrated edema, contrasting with glioblastoma’s pure edema.

**Supplementary Information:**

The online version contains supplementary material available at 10.1007/s00234-024-03385-0.

## Introduction

According to the latest World Health Organization (WHO) classification of CNS tumors, Isocitrate Dehydrogenase (IDH)-mutant astrocytoma grade 4 is no longer referred to as Glioblastoma. This term is now reserved exclusively for IDH-wildtype grade 4 astrocytic tumors [1]. A non-invasive differentiation of these grade 4 astrocytic tumors could have significant implications for patient management [2–7]. Astrocytoma grade 4 has been less extensively studied compared to its grade 2–3 counterparts, remaining a major radiological challenge. While lower-grade IDH-mutant astrocytomas typically appear as non-enhancing and non-necrotic on morphological images, grade 4 astrocytomas can mimic the imaging characteristics of glioblastomas. Both often exhibit enhancement and necrosis, contrasting starkly with their grade 2–3 IDH-mutant counterparts [7–13]. This overlap in morphological features between IDH-mutant and IDH-wildtype grade 4 tumors highlights the need for advanced quantitative MR techniques, such as Dynamic-Susceptibility-Contrast Perfusion-Weighted-Imaging (DSC-PWI).

DSC-PWI provides insights into the tumors’ vascular and microvascular environments [14], particularly relevant because microvascular proliferation is a defining feature of grade 4 according to the 2021 WHO classification [1]. As a result, predictably, both of these tumors, regardless of IDH status, should exhibit elevated relative cerebral-blood-volume (rCBV), which can be considered a radiological manifestation of microvascular proliferation. Thus, the question arises: Does high rCBV predict IDH-mutation status or merely denote a grade 4 tumor? Traditionally, rCBV calculations have focused on either mean or extreme values (maximum or “hot-spots”) derived from manually delineated regions-of-interest (ROIs) or entire tumor volumetric segmentations. The practice of using preselected single rCBV values, particularly when focused solely on specific ROIs, tends to overlook tumor heterogeneity, potentially overlooking significant differences across the entire tumor and spectrum of rCBV values [15, 16]. Furthermore, in these grade 4 tumors, both enhancing and non-enhancing components often coexist, which represent different tumor environments, and their separate evaluation could offer diverse perspectives [17].

Differentiating astrocytoma grade 4 and glioblastoma is especially crucial in patients under 55-year-old. According to WHO guidelines, DNA sequencing to confirm IDH mutation status in grade 4 astrocytic tumors is mandatory for patients under 55 years of age, while negative immunohistochemistry suffices for those above this age, given the rarity of IDH mutations beyond this threshold. Yet, for those under 55, IDH mutations are more balanced, emphasizing the need for accurate differentiation in this age group [1, 7, 18, 19]. Given the heavily correlated factors of age, grade, and IDH-mutation status, studies must approach these entities with care to ensure accurate representation of data and interpretation. Thus, focused studies are crucial for a deeper understanding.

Based on these rationales, we believe that the pre-surgical differentiation between IDH-mutant astrocytoma grade 4 and IDH-Wildtype Glioblastoma deserves specific attention [13]. The primary objective of this work is to study the potential of rCBV in distinguishing the IDH-mutation status of grade 4 astrocytic tumors in an age-adjusted cohort, in accordance with WHO recommendations regarding IDH-mutations [1, 18, 19]. We aim to asses both the enhancing and non-enhancing components in a comprehensive voxel-wise, automated, unsupervised manner (exploratory, without the input of prior knowledge or assumptions) using histogram-derived percentile values, contrasting with conventional methods that rely on preselected rCBV values such as mean or maximum.

## Methods

This retrospective study received approval from the Research Ethics Committee of our tertiary hospital.

### Patients

Patients diagnosed with IDH-mutant astrocytoma grade 4 and IDH-wildtype glioblastoma were retrospectively retrieved from our centre’s database spanning the years 2016–2023. The study’s inclusion criteria were as follows: (1) Confirmed tumor diagnosis in accordance with the WHO Classification of CNS Tumors 2021 criteria; (2) Age under 55-year-old at the time of tumor diagnosis, adhering to the WHO recommendations; and (3) Availability of a diagnostic pre-surgical MR imaging examination that includes DSC-PWI, T1WI, T2WI, FLAIR, and contrast-enhanced T1WI (CE-T1WI). The study’s exclusion criterion was the absence of any of the sequences or a low quality that prevented adequate tumor segmentation or DSC-PWI data extraction.

### Imaging

The MR imaging examinations included in the study were performed using a 1.5-T scanner (Ingenia, Philips Healthcare). All DSC-PWI sequences were gradient-echo, with the following technical parameters: Echo Time, 40ms; Repetition Time, 1500-1700ms; Flip- Angle, 75º; Pixel size, 1.75 mm; Slice Thickness, 5 mm; Image size, 128 × 128; Number of Slices, 20–25; Number and duration of Dynamics, 60 and 1.5s. A single dose of 0.1 mmol/kg of intravenous gadolinium-based contrast agent (1 mmol/mL) was injected at a rate of 4–5 mL/s. Baseline was in the order of 10–15 points. The quality of the sequences was assessed by two experienced neuroradiologists: A.P.-E and PN-B with more than 10 and 5 years of experience in neuroradiology. Examinations were labelled as poor quality and thus excluded from the study if: (1) motion artifacts prevented the segmentation or coregistration, or (2) an obvious low signal-to-noise ratio was visually assessable in the mean raw time-intensity curves.

### Post-processing and DSC-PWI data extraction

After following the standard recommended preprocessing steps, the HD-GLIO pipeline was utilized to segment both enhancing and non-enhancing regions of the whole brain tumor-related abnormality, considering axial T1WI, T2WI, FLAIR, and CE-T1WI [20, 21]. Necrosis was excluded. Subsequently, the FAST tool within FSL was employed to acquire the segmentation for normal-appearing white matter for normalization purposes [22]. Finally, the segmentations were co-registered with the DSC-PWI using the 3D Slicer BRAINSFit module (http://www.slicer.org). The segmentations were reviewed and verified by two experienced neuroradiologists: A.P-E. and PN-B. For each voxel within the tumor segmentations, normalized and leakage corrected rCBV was calculated as described by Boxerman et al. [14]. For each patient’s tumor segmentations, the mean and maximum values of all the voxels as well as percentile values in increments of five were calculated.

### Description and comparison of DSC-PWI metrics

Statistical comparisons were conducted for Grade 4 Astrocytoma and Glioblastoma rCBV mean, maximum and percentile values via a Mann-Whitney U test. Simultaneously, we calculated the area under the receiver operating characteristic curve (AUC-ROC) for all rCBV values. Finally, box-plots were constructed to visually assess the segregation potential of the different rCBV value.

As an addition, just to reinforce our observations that these two tumors display similar characteristics on morphological imaging, we referred to the most recent research [8–13]. According to these studies, the primary imaging markers for IDH-mutation status on morphological MRI might include nodular enhancement, necrosis, and T2-FLAIR mismatch. These markers were assessed dichotomously (for enhanced clarity, reproducibility and robustness) by two experienced radiologists (AP-E and PN-B), who determined the presence or absence of such signs.

## Results

### Patients

**Fig. 1:**
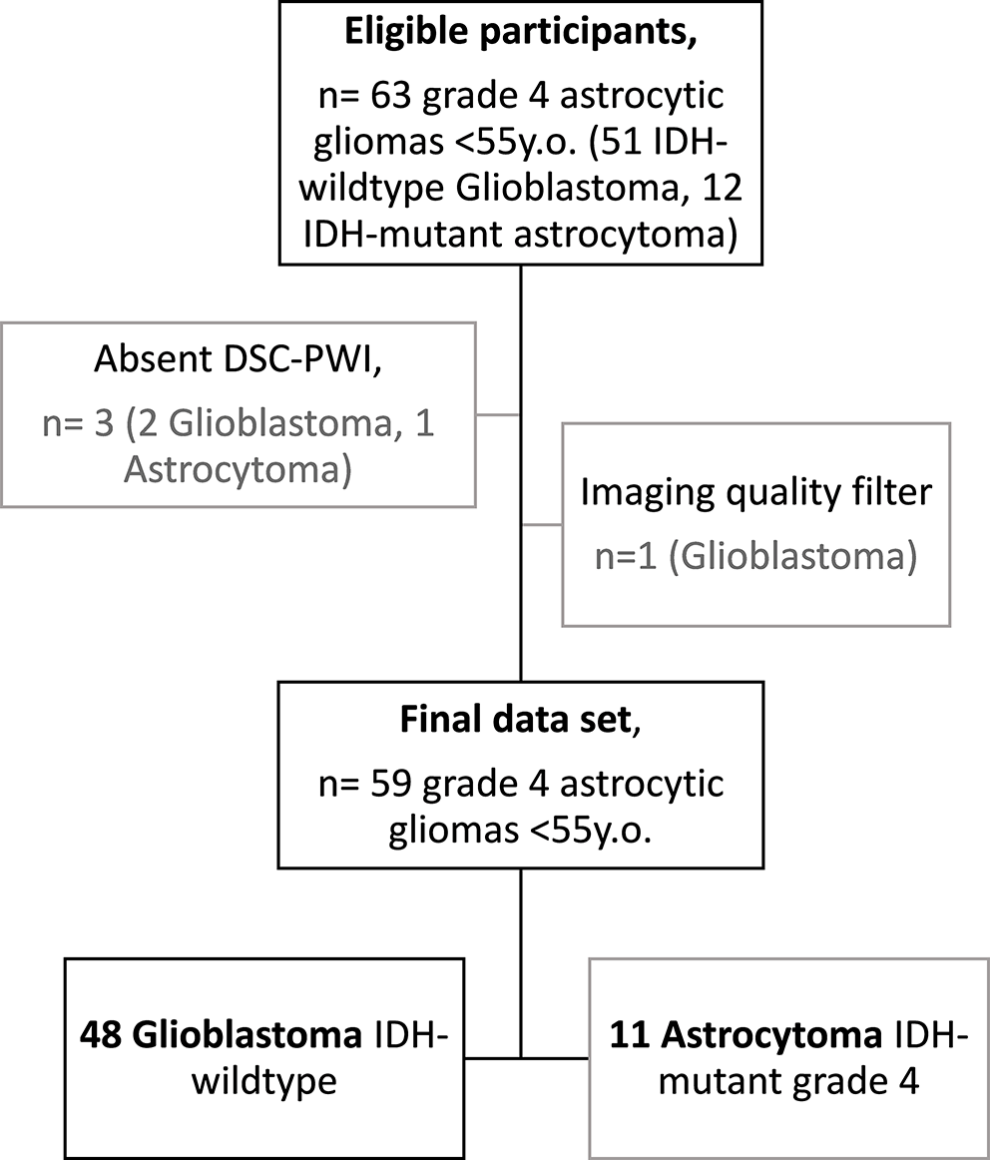
A flowchart that summarizes the study participant selection process

The initial cohort consisted of 63 grade 4 astrocytic tumors in patients under 55-year-old. This group was made up of 12 Astrocytoma grade 4 (Astrocytoma 4) and 51 Glioblastoma. Four tumors (1 Astrocytoma 4, and 3 Glioblastomas) were excluded due to the absence of DSC-PWI or the presence of motion artifacts that precluded accurate tumor segmentation or DSC-PWI data extraction. Consequently, the resulting dataset comprised 59 tumors: 11 Astrocytoma 4, and 48 Glioblastoma. A flowchart detailing the patient selection process is provided in Fig. 1.

Demographic details, including age and sex, are presented in Table 1. This table also highlights statistical comparisons between the two groups. The mean age across the dataset was 46-year-old, with 34 of the 59 participants being male. Despite the age-centric nature of the study, a significant age difference (*p* = 0.009) emerged between the entities, with Glioblastoma patients being slightly older, which aligns with prior knowledge [1]. Additionally, warranting particular mention and fully consistent with established knowledge, our reference centre’s brain tumor database did not include any Astrocytoma 4 in patients over 55-year-old.

**Table 1:** This table presents the demographic and clinical characteristics of the participants, including age, sex, and tumor grade

	Grade 4 astrocytoma IDH-Mutant	Glioblastoma IDH-wildtype	Whole data-set	*p*-value
Age (years), Mean +/-SD	41 +/-8	47 +/- 6	46 +/- 7	0.009*
Sex, Men: Women	6: 5	28: 20	34: 25	1
Total	11	48	59	

Statistical comparisons were made using the U-Mann Whitney Test for age, and the Chi-Square test for sex. SD denotes Standard Deviation.(*) indicates statistical significance, *p* < 0.05.

**Fig. 2:**
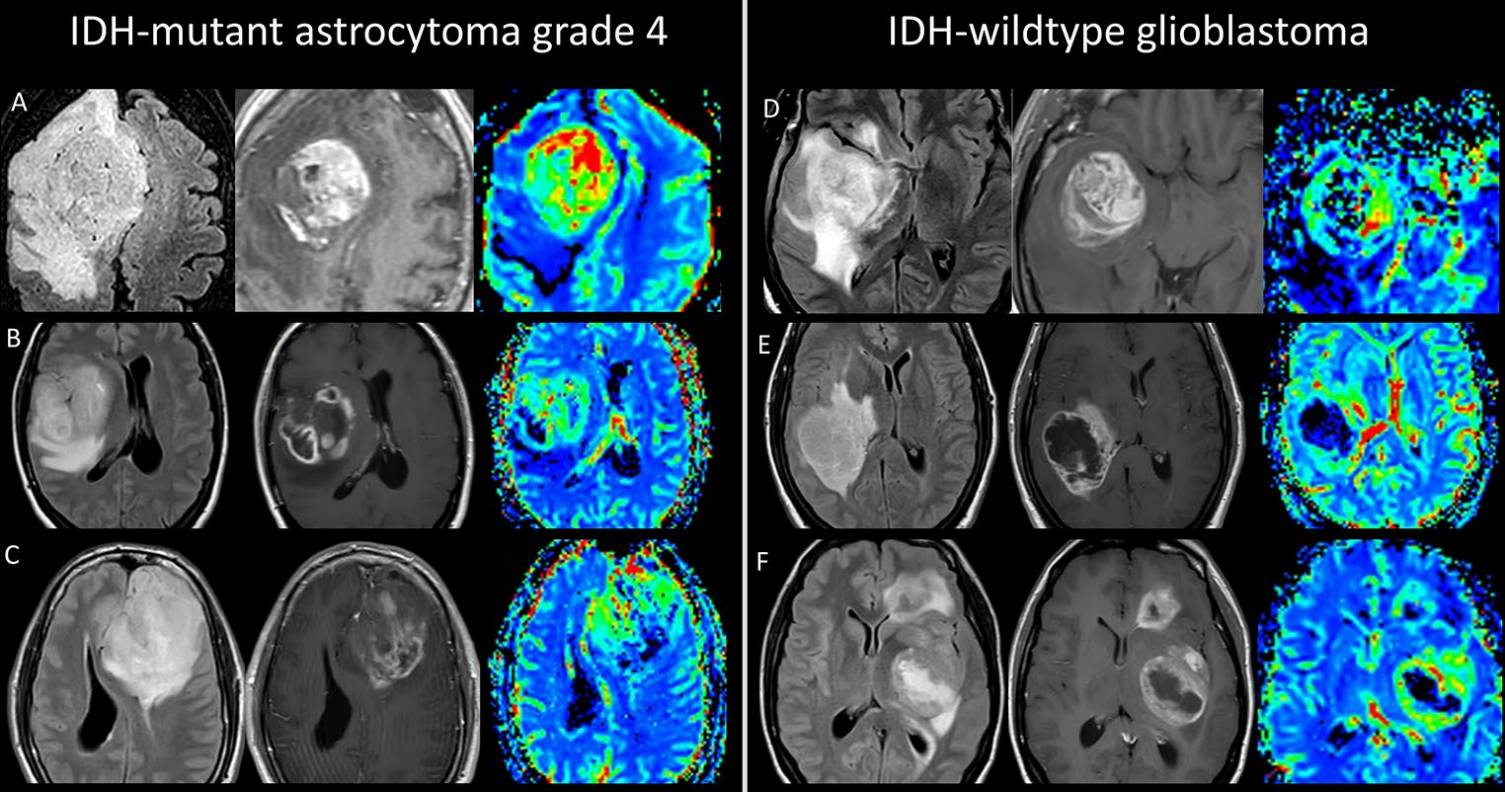
Illustrative cases of patients aged between 36 and 51 years diagnosed with Astrocytoma grade 4 (**A**-**C**) and Glioblastoma (**D**-**F**). These cases demonstrate overlapping imaging characteristics on morphological MR (FLAIR and CE-T1WI) and rCBV color maps. Features such as non-enhancing regions, nodular enhancements, conspicuous signs of necrosis, and high rCBV are common to both tumor types in all shown cases

Regarding morphological imaging evaluation (detailed in Table 2), the T2-FLAIR mismatch was the only visual feature showing significant differences associated with IDH-mutation status, boasting perfect specificity. However, it was observed in just 27% of the cases. No significant differences were observed in the presence or absence of nodular enhancement or necrosis, present in vast majority of cases for both entities as shown in Fig. 2.

**Table 2:** Distribution of presence of Nodular enhancement, necrosis and T2-FLAIR mismatch among tumor groups

Tumor type/ imaging features	Grade 4 astrocytoma IDH-Mutant	Glioblastoma IDH-wildtype	*p*-value
Nodular enhancement	9/11 (82%)	47/48 (98%)	0.16
Necrosis	9/11 (82%)	44/48 (92%)	0.52
T2-FLAIR mismatch	3/11 (27%)	0/48 (0%)	0.03*

Statistical comparisons were made using Chi-Square. (*) indicates statistical significance, *p* < 0.05.

### Description and comparison of DSC-PWI metrics

**Fig. 3:**
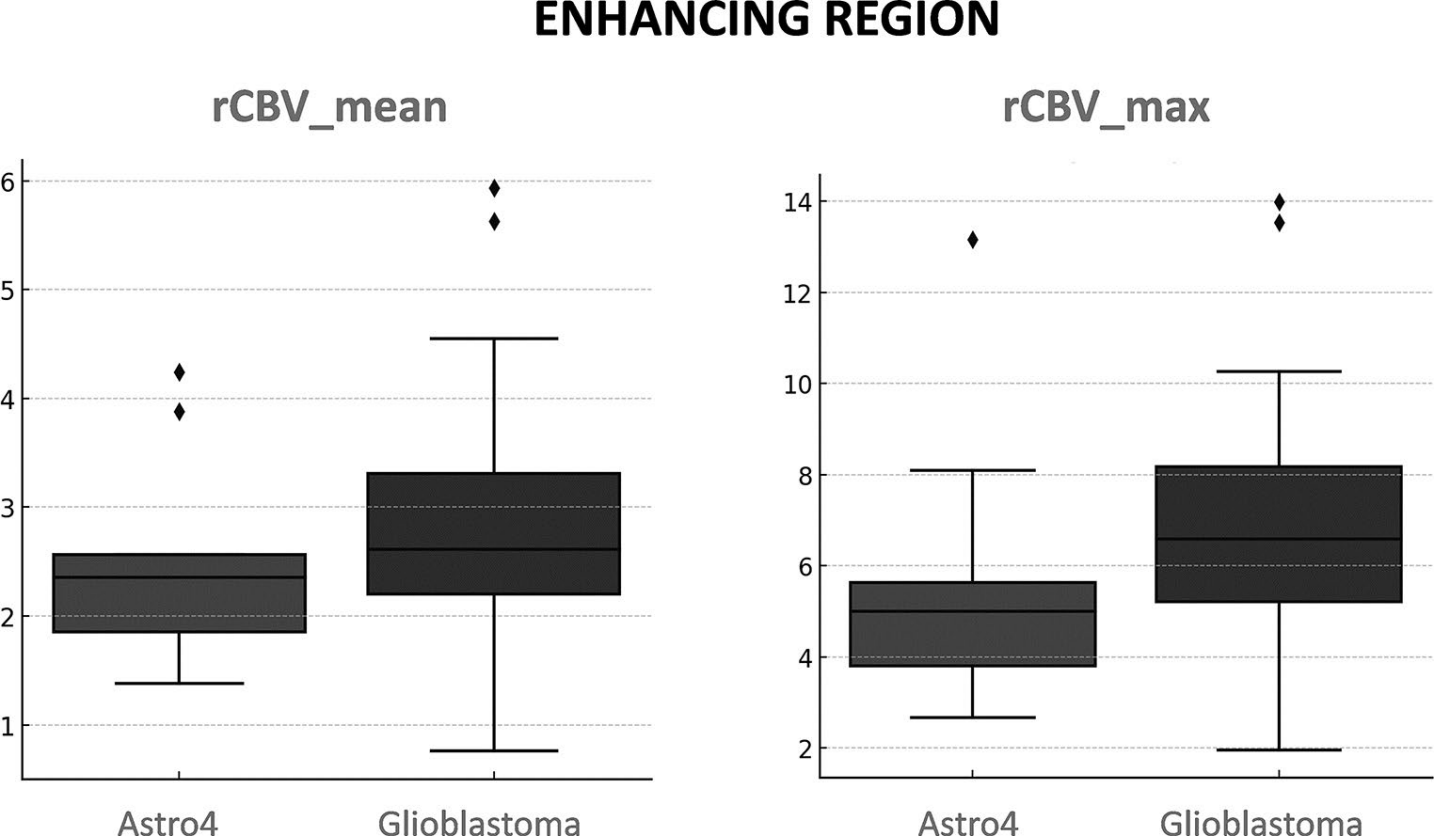
Whisker plots depict the distribution of mean and maximum rCBV values for the enhancing region of each tumor subtype. For clarity, only mean and maximum rCBV values are shown due to their widespread utilization in clinical practice, and also because none of the percentiles yielded significantly improved results

**Fig. 4:**
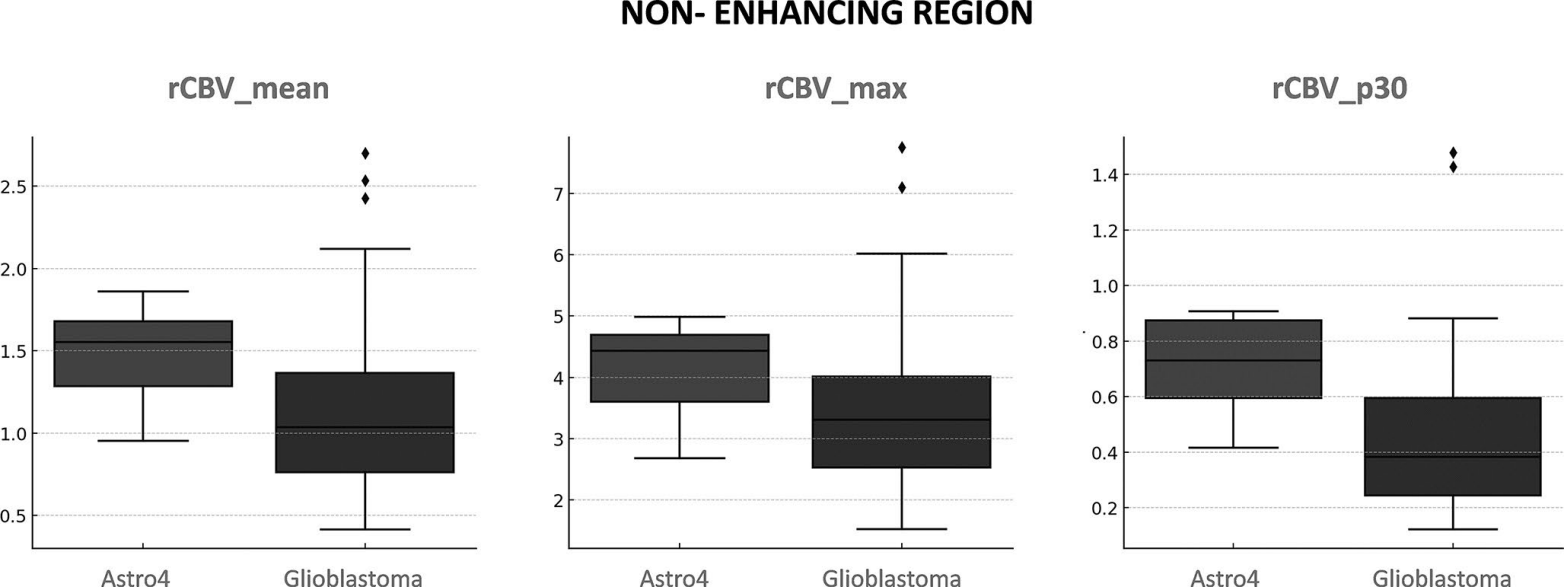
Whisker plots display the distribution of mean, maximum, and best percentile (p30) rCBV values for the non-enhancing region of each tumor subtype. For clarity, mean and maximum rCBV values are shown for comparison purposes because they are the most standard measures used in clinical practice. Meanwhile, p30 represents the best percentile result and surpasses those obtained with the standard approaches

Tables 3 and 4 present the results for rCBV comparisons using the U-Mann Whitney p-values and AUC-ROCs for enhancing and non-enhancing regions respectively. In the context of the enhancing tumor (Table 3), IDH-wildtype displayed higher values overall, but no significant differences emerged between the two tumor types. Interestingly, the lower percentiles (those between p5 and p35) in enhancing regions showed higher rCBV values in IDH-mutant, also no significant. For the non-enhancing regions (Table 4), all rCBV percentile values exhibited significant differences between both entities, using the conventional p-value threshold of 0.05. Overall rCBV in non-enhancing regions were higher in Astrocytomas 4. Applying the more stringent threshold of *p* < 0.005, recently proposed as more robust [23], the mean rCBV (Astrocytoma = 1.48 vs. Glioblastoma = 1.14) and percentile rCBV values between p10 and p60 (Astrocytoma = 0.35–1.32 vs. Glioblastoma = 0.21–0.96) remain significant. In AUC-ROC analysis, excellent discriminatory power considered as above 0.8 [24], was demonstrated by the percentiles between p15 and p30. Whisker-plots for the mean and maximum rCBV of enhancing region are shown in Fig. 3. The plots for non-enhancing region mean, maximum, and the best percentile (p30) rCBV values are shown in Fig. 4. These figures allow for a visual assessment of the different discriminative capabilities and values dispersion. The sensitivity and specificity of rCBVp30, the best percentile, were 0.82 and 0.71, respectively, for a threshold of 0.56.

**Table 3:** Average rCBV values (mean, maximum, and percentiles) for both tumor types in enhancing regions

Enhancing region								
Astro 4	rCBV	Mean	Gb	rCBV	Mean		*p*	AUC-ROC
	rCBV mean	2.496		rCBV mean	2.784		0.315	0.608
	rCBV p5	0.638		rCBV p5	0.359		0.074	0.690
	rCBV p10	0.901		rCBV p10	0.648		0.103	0.674
	rCBV p15	1.074		rCBV p15	0.894		0.255	0.622
	rCBV p20	1.275		rCBV p20	1.117		0.360	0.598
	rCBV p25	1.454		rCBV p25	1.321		0.475	0.577
	rCBV p30	1.577		rCBV p30	1.513		0.672	0.546
	rCBV p35	1.715		rCBV p35	1.707		0.947	0.508
	rCBV p40	1.860		rCBV p40	1.902		1.000	0.501
	rCBV p45	2.009		rCBV p45	2.106		0.841	0.522
	rCBV p50	2.157		rCBV p50	2.322		0.672	0.546
	rCBV p55	2.300		rCBV p55	2.539		0.577	0.560
	rCBV p60	2.471		rCBV p60	2.777		0.422	0.586
	rCBV p65	2.649		rCBV p65	3.045		0.315	0.608
	rCBV p70	2.830		rCBV p70	3.337		0.237	0.626
	rCBV p75	3.043		rCBV p75	3.700		0.160	0.650
	rCBV p80	3.373		rCBV p80	4.122		0.147	0.655
	rCBV p85	3.812		rCBV p85	4.659		0.129	0.662
	rCBV p90	4.617		rCBV p90	5.417		0.141	0.657
	rCBV max	5.772		rCBV max	6.763		0.090	0.681

Statistical comparisons were conducted using the U-Mann Whitney Test and AUC-ROC. (*) indicates statistical significance, *p* < 0.05 and/or AUC-ROC > 0.8. (**) indicates statistical significance, *p* < 0.005.

**Table 4:** Average rCBV values (mean, maximum, and percentiles) for both tumor types in non-enhancing regions

Non-enhancing region								
Astro 4	rCBV	Mean	Gb	rCBV	Mean		*p*	AUC-ROC
	rCBV mean	1.484		rCBV mean	1.137		0.004**	0.782
	rCBV p5	0.204		rCBV p5	0.122		0.053	0.689
	rCBV p10	0.353		rCBV p10	0.205		0.003**	0.792
	rCBV p15	0.449		rCBV p15	0.270		0.002**	0.801*
	rCBV p20	0.535		rCBV p20	0.335		0.002**	0.807*
	rCBV p25	0.621		rCBV p25	0.396		0.001**	0.811*
	rCBV p30	0.705		rCBV p30	0.458		0.001**	0.811*
	rCBV p35	0.794		rCBV p35	0.526		0.002**	0.799
	rCBV p40	0.881		rCBV p40	0.590		0.002**	0.797
	rCBV p45	0.979		rCBV p45	0.662		0.003**	0.79
	rCBV p50	1.082		rCBV p50	0.745		0.002**	0.795
	rCBV p55	1.194		rCBV p55	0.846		0.003**	0.792
	rCBV p60	1.321		rCBV p60	0.957		0.004**	0.784
	rCBV p65	1.459		rCBV p65	1.084		0.006*	0.771
	rCBV p70	1.623		rCBV p70	1.236		0.008*	0.759
	rCBV p75	1.832		rCBV p75	1.420		0.008*	0.759
	rCBV p80	2.091		rCBV p80	1.652		0.015*	0.739
	rCBV p85	2.434		rCBV p85	1.972		0.020*	0.727
	rCBV p90	2.985		rCBV p90	2.498		0.022*	0.723
	rCBV max	4.080		rCBV max	3.440		0.025*	0.72

Statistical comparisons were conducted using the U-Mann Whitney Test and AUC-ROC. (*) indicates statistical significance, *p* < 0.05 and/or AUC-ROC > 0.8. (**) indicates statistical significance, *p* < 0.005.

To clarify, for example, p5 corresponds to the rCBV value below which 5% of voxels within the segmented volume-of-interest fall, meaning 95% are above it, while p10 is the value below which 10% of voxels fall, meaning 90% are above it, and so forth. Therefore, for instance the range from p5 to p35 represents the lower range of rCBV values within the segmented volume-of-interest, from the 5th percentile to the 35th percentile, with values above the lowest 5% and below the highest 65%. For clarity and transparency, we provide the full list of results for all percentiles in both tumor regions. This approach offers an exploratory alternative without relying on prior knowledge or assumptions, and studying all percentiles ensures full data availability and the robustness of the provided information.

Two additional subanalyses were conducted to reinforce the main findings: one involved a class-balanced 5-fold internal cross-validation to address potential class imbalance (11 Astrocytoma: 48 Glioblastoma) biases, and the other applied Bonferroni corrections to p-values. These are detailed in Supplementary Materials 1 and 2, respectively. In these analyses, the lower percentiles of non-enhancing regions remained significant and exhibited stable AUC-ROC values under very stringent conditions, whereas the mean and maximum values lost their significance. This confirms our main results, highlighting not only the superior performance of lower percentiles in the non-enhancing regions but also their greater veracity, robustness and stability compared to the mean and maximum values. For additional comparison of the main rCBV variables, an additional figure of dispersion graphics is also provided in Supplementary Material 3.

## Discussion

In this study, we assessed the discriminatory potential of rCBV derived from DSC-PWI to non-invasively differentiate between IDH-mutant astrocytoma grade 4 and IDH-wildtype glioblastoma pre-surgically. Our voxel-wise approach, which accounted for volumetric segmentations and all percentile values, revealed that the most discriminative rCBV values lie within the lower percentiles of the non-enhancing regions. Here, though the values are overall low, they are notably higher in IDH-mutant tumors, suggesting the benefit of using an unsupervised rCBV selection approach over the conventional reliance on preselected mean or maximum values.

Furthermore, given the well-known coexistence of tumor infiltration and edema in the non-enhancing regions, we propose that these differential rCBV values may stem from varying degrees of tumor infiltration in these low-vascularized non-enhancing areas. Such regions may represent a greater degree of coexisting very-low vascularized infiltrated tissue in IDH-mutant cases, while in IDH-wildtype cases, they may more closely align with pure edema. Indeed, this hypothesis aligns well with prior knowledge: Glioblastomas are known to generate more pronounced edema, whereas Astrocytomas manifest a more substantial proportion of non-enhancing tumor tissue in the T2-FLAIR abnormality [25].

The observed elevated rCBV values in enhancing regions in both tumors, with no significant differences between them, would support the hypothesis that microvascular proliferation is a characteristic of grade 4 tumors, rather than a specific attribute of IDH-mutation status. Interestingly, the lower percentile rCBV values for Astrocytoma grade 4 tend to be slightly higher than those for Glioblastoma within these enhancing regions. This trend may suggest a higher homogeneity in Astrocytomas, characterized by a narrower range of rCBV values when compared to Glioblastomas.

Grade 4 Astrocytomas present morphological imaging traits that are distinct from grade 2–3 but are more reminiscent of IDH-wildtype Glioblastoma. The challenge of radiologically distinguishing between these two entities is highlighted by the morphological evaluations in this study, as illustrated in Fig. 2; Table 2, when considering the main markers described for differentiating between IDH-mutant and IDH-wildtype tumors [8–13].

**Fig. 5:**
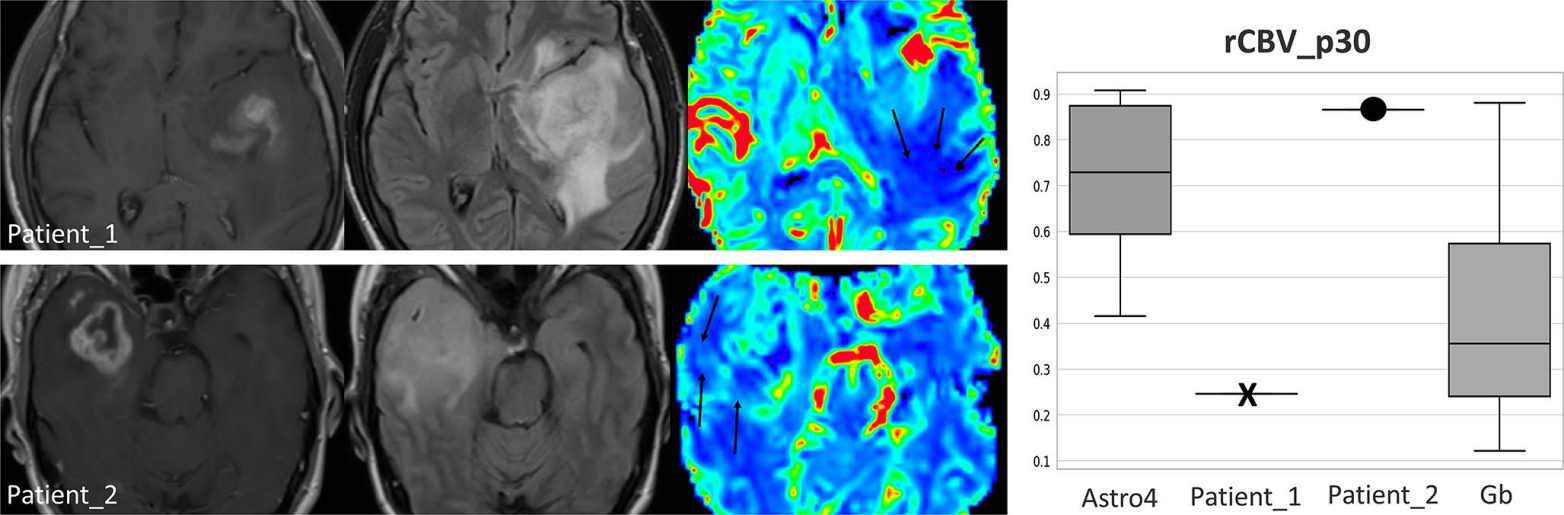
Illustrative cases of two patients with unknown diagnosis: Patient_1 is 51 years old, and Patient_2 is 49 years old. The images display an extensive non-enhancing component beyond the enhancing tumor margins. This could be attributed to infiltrative tumor, edema, or a coexistence of both. rCBV color maps focused analysis allow the detection of small foci of slightly elevated rCBV (arrows) in the non-enhancing component of Patient_2, while it depicts clear areas of very low rCBV (arrows) in Patient_1. Quantification of the 30th percentile in non-enhancing areas indicates that Patient_1 has values that fall within the range of Glioblastoma (Gb), while Patient_2 aligns with Astrocytoma grade 4 (Astro 4). The diagnoses for both cases were histopathology confirmed

Additionally, this study underscores the clinical significance of this differentiation in patients under 55 years old. In realistic clinical settings, the differentiation becomes crucial in this age group, making our findings especially pertinent. Unlike in those over 55 where the prevalence of the IDH mutation is negligible [1, 18, 19]. This approach mirrors a real-world clinical scenario where such differentiation is genuinely pertinent and impactful. For instance, the non-invasive presurgical differentiation of grade 4 astrocytic tumors is relevant beyond the ultimate histopathological diagnosis and could profoundly impact patient management across different levels. First, in specific scenarios, it could influence surgical decisions, such as whether to opt for function-preserving surgery or a biopsy (in cases of suspected grade 4 astrocytomas) versus total resection (in cases of suspected glioblastomas), particularly in challenging locations. Second, it may guide the sequence of the diagnostic workflow in histopathology and molecular pathology. For instance, by emphasizing and optimizing DNA sequencing utilization (often costly or difficult to access) in the most indicated cases to optimally detect IDH mutations. Ultimately, it offers an early prognosis prediction, which is invaluable, especially for young adults, and their families, enabling informed decisions and setting realistic expectations. Furthermore, such differentiation could be instrumental for the early detection of clinical trial candidates, for instance, for trials on treatments targeting IDH, which are anticipated to increase due to recent positive outcomes [2]. As we move further into the era of personalized and targeted therapies, the insights from our study could play an increasingly important role in shaping treatment strategies. This, in turn, hopefully will positively influence the disease course and enhance the quality of life for patients [3–6]. An illustrative example of potential clinical applicability of results in new patients with unknown diagnosis is shown in Fig. 5. Four additional illustrative cases are provided in Supplementary Material 4, along with the rCBVp30 values for the entire dataset.

Several studies have attempted to identify IDH-mutation status using rCBV while analysing a range of adult diffuse gliomas. Some suggest the feasibility of discerning IDH mutation status, generally reporting higher rCBV values in both enhancing and non-enhancing regions for IDH-wildtype [17, 26, 27]. However, interpreting these findings requires caution, as these studies do not account for potential confounding with age or histological grade which are only reported as descriptive statistics, thereby preventing the optimal discernment of the specific differential in the current study. As an exemplification, considering that the vast majority of grade 4 astrocytic tumors are indeed glioblastomas, and the vast majority of grade 2–3 are IDH-mutant astrocytomas, a study claiming to identify IDH mutation status might actually be reflecting a more familiar differentiation between grade 2–3 and grade 4. Lastly, it is crucial to recognize that astrocytoma grade 4 is often either absent or significantly underrepresented in such studies, which limits the applicability of their results to this specific, smaller subgroup. This subgroup necessitates particular attention, as provided in our study.

Our literature search yielded only two DSC-PWI studies explicitly focused on grade 4 astrocytic tumors [28, 29], which in general terms reported higher rCBV values in IDH-wildtype tumors. However, due to different methodological approaches, direct comparison of results is not feasible. We consider relevant strengths of our methodology to include volumetric segmentations of easily demarcated morphological MR main tumor regions, which provide information on the entire abnormality; and the comprehensive evaluation of voxel-wise rCBV values through percentile analysis, not limited to preselected mean or maximum, which may obscure relevant differences in other parts of the full range of values.

Finally, another advanced MR technique deserving mention in this scenario is MR spectroscopy. It has been proven useful for IDH-mutation identification through specifically edited sequences, achieving high accuracies [30]. Also, more standard MR spectroscopy protocols offer information for glioma classification under the latest WHO guidelines [31]. However, the specific focused performance in grade 4 astrocytic tumors remains less clear because existing research again mixes tumor grades 2, 3, and 4. A potential limitation of this technique is its less extended implementation and use in neuroradiology departments worldwide compared to the widely extended and accepted DSC-PWI for brain tumor imaging [14, 15, 32, 33]. At any instance, recognizing the challenges, we believe that an ideal approach for the near future would combine comprehensive imaging data, including DSC-PWI and MR spectroscopy, with advanced data analysis techniques, such as AI and radiomics, to enhance presurgical tumor classification.

This study comes with several limitations. This is a single-site retrospective investigation. Nevertheless, this approach ensured data homogeneity, useful in pilot studies. The sample size, though seeming modest, is justified as all tumors were classified based on the stringent 2021 WHO Classification criteria, limiting retrospective patient inclusion. Also, IDH-mutant grade 4 astrocytomas are infrequent tumors, and they are rarely addressed in recent literature as a separate entity from their grade 2–3 counterparts. We recognize that theoretically, preloaded or low Flip-Angle (30º) DSC-PWI sequences might optimize rCBV measures when aligned with histological vascularization evaluations [14]. Yet, the primary differences lie in the non-enhancing region of tumors, where leakage-effects due to blood-brain-barrier disruption should be negligible, thus reducing the impact on rCBV calculations. Moreover, our study’s main focus wasn’t solely on this alignment. Different techniques have also shown reproducibility and robustness and we applied rigorous leakage correction procedures, mitigating potential leakage impacts [34]. Additionally, it should be highlighted that many clinicians have a preference for non-preloaded intermediate-high Flip-Angle sequences, particularly when it comes to the pre-surgical differential diagnosis [35–41]. This preference aligns with our study’s context and has demonstrated to be useful for diffuse gliomas’ genetic subtypes presurgical differentiation [42, 43]. However, our methodology can adapt to different DSC-PWI techniques with simple threshold adjustments [44]. Nevertheless, broader multicentric validations remain essential. Lastly, unfortunately, analysing a single case using our proposed methodology currently requires 10–15 min, hindered by the limitations of commercial software in PACS systems that force the use of multiple tools. This situation could impede rapid clinical adoption, but also highlights an opportunity for software enhancement in clinical neuroradiology, especially through improved segmentation tools and presenting CBV values via percentile analysis. With these improvements, post-processing time could potentially be reduced to around 2 min, underscoring the need for software advancements to narrow the gap between clinical practice and research in neuroradiology.

On the other hand, our study’s strengths are evident. All tumors were rigorously classified as per the 2021 WHO Classification criteria, ensuring contemporaneity. The tumor groups have been carefully balanced accounting for grade and age. Our insights hold clinical relevance from multiple discussed vantage points. Notably, the automatization of the data-extraction and data-selection ensures reproducibility minimizing operator-dependency. We underscore the importance of an unsupervised evaluation of the tumors’ entire percentile values, challenging the common clinical practice of relying on ROIs, mean or maximum values. In essence, our findings could be extrapolated to other clinical scenarios, laying the groundwork for further research.

## Conclusion

Using an unsupervised percentile-based approach to select rCBV values from volumetric segmentations provides richer information compared to the use of ROIs and preselected mean and maximum values.

The non-enhancing components of grade 4 astrocytic tumors are potentially more informative than the enhancing region itself.

Although globally low, higher rCBV values in the lower percentiles of the non-enhancing region in IDH-mutant grade 4 astrocytomas rose as the most distinguishing feature from glioblastomas. This suggests that different proportions of infiltrative versus vasogenic edema might be a clue for differentiating these two tumor types.

### Electronic supplementary material

Below is the link to the electronic supplementary material.

Supplementary Material 1

## Abbreviations

DSC-PWI: Dynamic-Susceptibility-Contrast Perfusion-Weighted-Imaging
rCBV: Relative Cerebral Blood Volume
IDH: Isocitrate Dehydrogenase
WHO: World Health Organization
ROI: Region Of Interest
AUC-ROC: Area Under the Receiver Operating Characteristic Curve
