# Supplementary material for: DSC-PWI presurgical differentiation of grade 4 astrocytoma and glioblastoma in young adults: rCBV percentile analysis across enhancing and non-enhancing regions
Source: Neuroradiology. 2024 Jun 5;66(8):1267–77. doi: 10.1007/s00234-024-03385-0 (PMC11246293; doi:10.1007/s00234-024-03385-0)
Supplement: Supplementary file 1 — Supplementary Material 1 [file 234_2024_3385_MOESM1_ESM.pdf]

SUPPLEMENTARY MATERIAL

DSC-PWI Presurgical Differentiation of Grade 4 Astrocytoma and Glioblastoma in Young Adults: rCBV Percentile Analysis Across Enhancing and Non-Enhancing Regions

Albert Pons-Escoda1-3, Pablo Naval-Baudin1,3,4, Mildred Viveros1, Susanie Flores-Casaperalta1, Ignacio Martinez-Zalacaín1,4, Gerard Plans5,2, Noemi Vidal6,2, Monica Cos1, Carles Majos1,2

1. Radiology Department, Hospital Universitari de Bellvitge, Barcelona, Spain
2. Neuro-oncology Unit, Institut d'Investigació Biomèdica de Bellvitge- IDIBELL, Barcelona, Spain
3. Facultat de Medicina i Ciències de La Salut, Universitat de Barcelona (UB), Barcelona, Spain
4. Diagnostic Imaging and Nuclear Medicine Research Group, Institut d'Investigació Biomèdica de Bellvitge- IDIBELL, Barcelona, Spain
5. Neurosurgery Department, Hospital Universitari de Bellvitge, Barcelona, Spain
6. Pathology Department, Hospital Universitari de Bellvitge, Barcelona, Spain

Corresponding author:

Albert Pons-Escoda,

albert.pons@bellvitgehospital.cat

https://orcid.org/0000-0003-4167-8291

Supplementary material 1.

Results from the subanalysis utilizing 5-fold internal cross-validation (CV) to adress potential class imbalance biases.

ENHANCING REGION

Variable	CV p-Value Mean	CV p-Value SD	CV AUC-ROC Mean	CV AUC-ROC SD	p-Value (full dataset)	AUC-ROC (full dataset)
**rcbv mean**	0.580	0.389	0.608	0.119	0.315	0.608
**rcbv p5**	0.231	0.219	0.690	0.073	0.074	0.690
**rcbv p10**	0.295	0.294	0.673	0.086	0.103	0.674
**rcbv p15**	0.365	0.241	0.620	0.108	0.255	0.622
**rcbv p20**	0.370	0.138	0.596	0.109	0.360	0.598
**rcbv p25**	0.429	0.140	0.576	0.106	0.475	0.577
**rcbv p30**	0.520	0.135	0.545	0.100	0.672	0.546
**rcbv p35**	0.630	0.240	0.507	0.105	0.947	0.508
**rcbv p40**	0.646	0.279	0.503	0.112	1.000	0.501
**rcbv p45**	0.730	0.355	0.525	0.115	0.841	0.522
**rcbv p50**	0.666	0.345	0.548	0.128	0.672	0.546
**rcbv p55**	0.677	0.361	0.562	0.126	0.577	0.560
**rcbv p60**	0.668	0.388	0.588	0.117	0.422	0.586
**rcbv p65**	0.577	0.347	0.609	0.119	0.315	0.608
**rcbv p70**	0.510	0.337	0.628	0.125	0.237	0.626
**rcbv p75**	0.412	0.286	0.651	0.120	0.160	0.650
**rcbv p80**	0.383	0.269	0.655	0.111	0.147	0.655
**rcbv p85**	0.337	0.231	0.662	0.096	0.129	0.662
**rcbv p90**	0.333	0.234	0.657	0.080	0.141	0.657
**rcbv max**	0.250	0.173	0.681	0.073	0.090	0.681

NON-ENHANCING REGION

Variable	CV p-Value Mean	CV p-Value SD	CV AUC-ROC Mean	CV AUC-ROC SD	p-Value (full dataset)	AUC-ROC (full dataset)
**rcbv mean**	0.089	0.123	0.782	0.107	0.004	0.782
**rcbv p5**	0.251	0.322	0.686	0.095	0.053	0.689
**rcbv p10**	***0.048****	0.058	0.791	0.068	0.003	0.792
**rcbv p15**	***0.039****	0.037	0.801	0.070	0.002	0.801
**rcbv p20**	***0.041****	0.046	0.806	0.080	0.002	0.807
**rcbv p25**	***0.050****	0.065	0.810	0.097	0.001	0.811
**rcbv p30**	***0.040****	0.046	0.811	0.082	0.001	0.811
**rcbv p35**	***0.050****	0.065	0.800	0.091	0.002	0.799
**rcbv p40**	***0.050****	0.063	0.798	0.086	0.002	0.797
**rcbv p45**	0.063	0.074	0.791	0.094	0.003	0.790
**rcbv p50**	0.058	0.067	0.797	0.096	0.002	0.795
**rcbv p55**	0.069	0.083	0.793	0.103	0.003	0.792
**rcbv p60**	0.075	0.087	0.785	0.103	0.004	0.784
**rcbv p65**	0.111	0.142	0.771	0.119	0.006	0.771
**rcbv p70**	0.147	0.196	0.760	0.128	0.008	0.759
**rcbv p75**	0.144	0.184	0.760	0.128	0.008	0.759
**rcbv p80**	0.178	0.226	0.739	0.122	0.015	0.739
**rcbv p85**	0.220	0.322	0.726	0.127	0.020	0.727
**rcbv p90**	0.181	0.229	0.722	0.101	0.022	0.723
**rcbv max**	0.162	0.161	0.718	0.089	0.025	0.720

Specifically, this 5-fold internal cross-validation approach involved maintaining a fixed set of 11 Astrocytoma grade 4 samples and comparing them against balanced subsets of Glioblastoma samples (10 samples in 3 folds and 9 samples in 2 folds). Then, the

Mann-Whitney U test statistics and AUC-ROC values for each fold were calculated and analysed. The results from these folds were averaged to provide mean and standard deviation metrics.

Of note, the AUC-ROC values remained stable across the 5-fold cross-validation, with mean AUC-ROC values nearly identical to those derived from the full dataset for both enhancing and non-enhancing regions. As anticipated, the p-values were higher due to the reduced sample size and consequent decrease in statistical power. However, it is particularly noteworthy that the lower percentiles of the non-enhancing regions retained significance (p10 to p40 $p \leq 0.05$) even under these extremely stringent conditions. On the other hand, the mean, maximum and other percentiles did not maintain significance when the dataset was reduced in these folds. This highlights the greater veracity, stability and robustness of our results, particularly in the lower percentiles, underscoring that differences in these percentiles are not only more pronounced but also more stable and less variable due to sample variations compared to commonly used metrics like mean and maximum values. This subanalysis does not significantly alter the main findings of our paper but serves as a valuable addition, reinforcing the performance of lower percentiles in non-enhancing regions and effectively addressing concerns about potential class imbalance.

Supplementary material 2.

Additional results of U-Mann Whitney test p-values after Bonferroni correction.

ENHANCING REGION				NON-ENHANCING REGION		
**Variable**	**p-Value**	**p-Value (Corrected)**		**Variable**	**P-Value**	**p-Value (Corrected)**
**rcbv mean**	0.315	1.000		**rcbv mean**	0.004	0.077
**rcbv p5**	0.074	1.000		**rcbv p5**	0.053	1.000
**rcbv p10**	0.103	1.000		**rcbv p10**	0.003	0.056
**rcbv p15**	0.255	1.000		**rcbv p15**	0.002	***0.041****
**rcbv p20**	0.360	1.000		**rcbv p20**	0.002	***0.033****
**rcbv p25**	0.475	1.000		**rcbv p25**	0.001	***0.029****
**rcbv p30**	0.672	1.000		**rcbv p30**	0.001	***0.029****
**rcbv p35**	0.947	1.000		**rcbv p35**	0.002	***0.043****
**rcbv p40**	1.000	1.000		**rcbv p40**	0.002	***0.046****
**rcbv p45**	0.841	1.000		**rcbv p45**	0.003	0.060
**rcbv p50**	0.672	1.000		**rcbv p50**	0.002	***0.049****
**rcbv p55**	0.577	1.000		**rcbv p55**	0.003	0.056
**rcbv p60**	0.422	1.000		**rcbv p60**	0.004	0.072
**rcbv p65**	0.315	1.000		**rcbv p65**	0.006	0.111
**rcbv p70**	0.237	1.000		**rcbv p70**	0.008	0.158
**rcbv p75**	0.160	1.000		**rcbv p75**	0.008	0.158
**rcbv p80**	0.147	1.000		**rcbv p80**	0.015	0.292
**rcbv p85**	0.129	1.000		**rcbv p85**	0.020	0.401
**rcbv p90**	0.141	1.000		**rcbv p90**	0.022	0.444
**rcbv max**	0.090	1.000		**rcbv max**	0.025	0.492

These results also strengthen the findings from the main analysis, demonstrating that the lower percentiles (p15 to p40, and p50) of non-enhancing regions are not only significant with the strict p-value threshold of <0.005 used in the main results, but also remain significant even after applying a (probably exceedingly) stringent Bonferroni correction. In contrast, mean and maximum values, lose their significance after the correction. This additional evidence reinforces the veracity and the superior robustness and stability of the lower percentile results in non-enhancing regions for differentiating between the conditions studied.

Supplementary material 3.

Uniformly scaled dispersion graphics of rCBV mean, maximum, and p30 for additional visual assessments of differences.

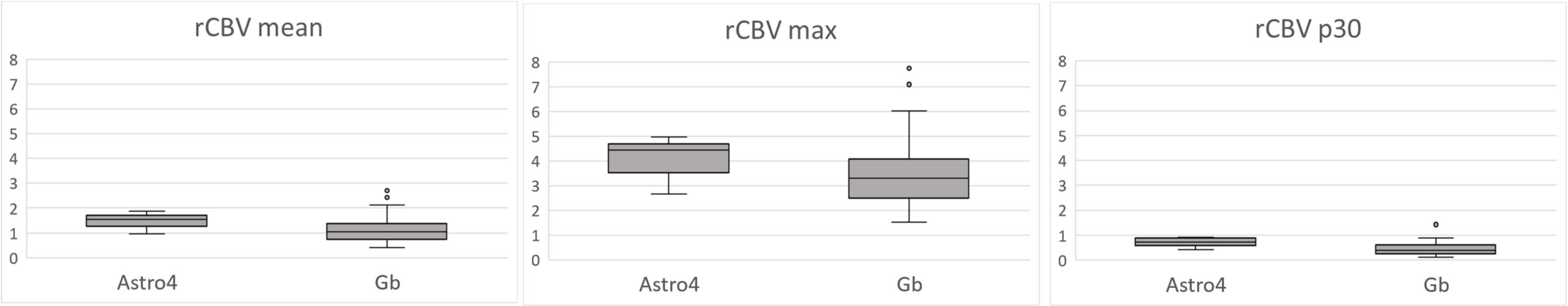

The Supplementary material 1 and 2 highlights that lower percentiles not only exhibit lower p-values and higher AUC-ROC values but also demonstrate greater robustness and stability compared to mean and maximum values when subjected to subanalysis. To facilitate comparisons, we hereby provide uniformly scaled graphics for the main variables of rCBV in non-enhancing regions (rCBV mean, maximum and p30) allowing additional insights through visual analysis. The differential robustness and stability is also visually evident from the graphs, where the range of values for percentiles is notably narrower than for mean and significantly more so than for maximum values, which show high variability.

Supplementary material 4.

Four additional illustrative cases of patients with unknown diagnosis: The images display extensive non-enhancing components beyond the enhancing tumor margins that could be attributed to infiltrative tumor, edema, or a coexistence of both. rCBV color maps focused analysis allow the detection of small foci of slightly elevated rCBV in the non-enhancing component of Patient_4 and _6, while it depicts clear areas of very low rCBV in Patient_3 and _5. Quantification of the 30th percentile in non-enhancing areas indicates that Patients_3 and _5 has values that fall within the range of Glioblastoma (Gb), while Patients_4 and _6 aligns with Astrocytoma grade 4 (Astro 4). The diagnoses for the four cases were histopathology confirmed.

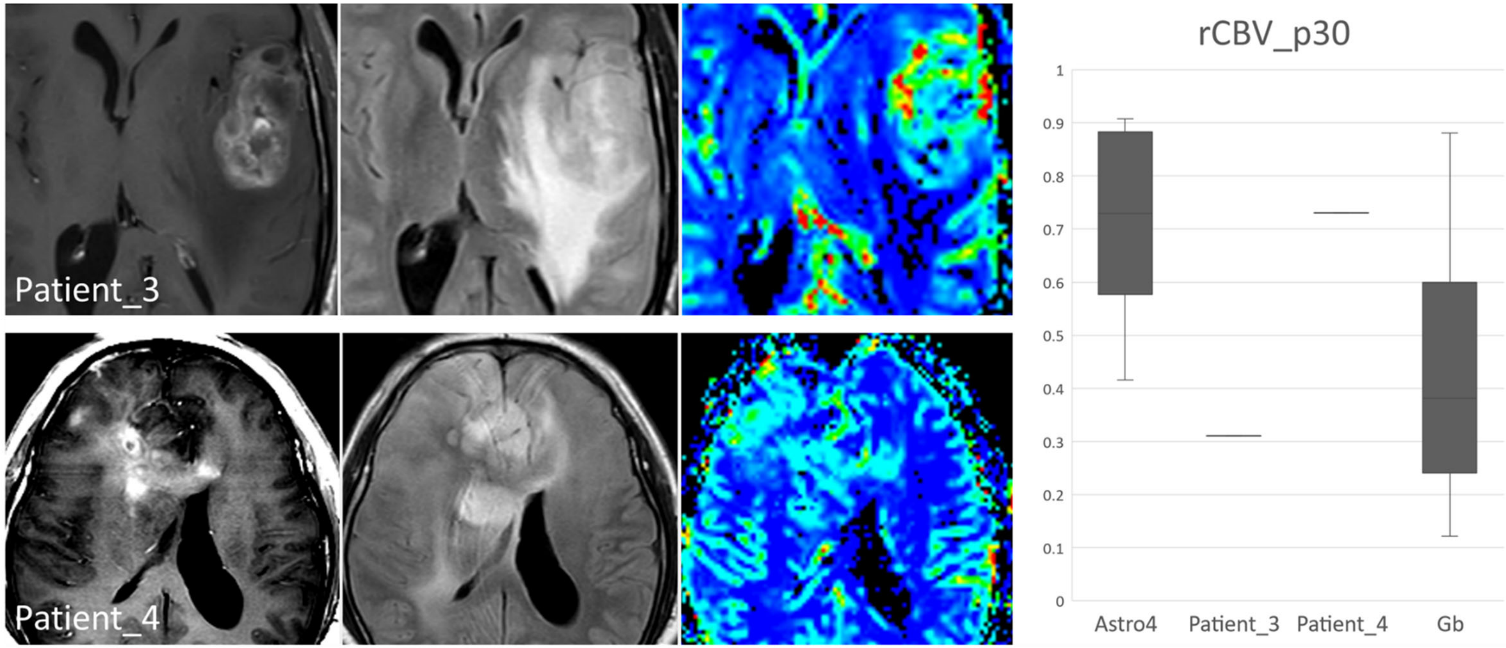

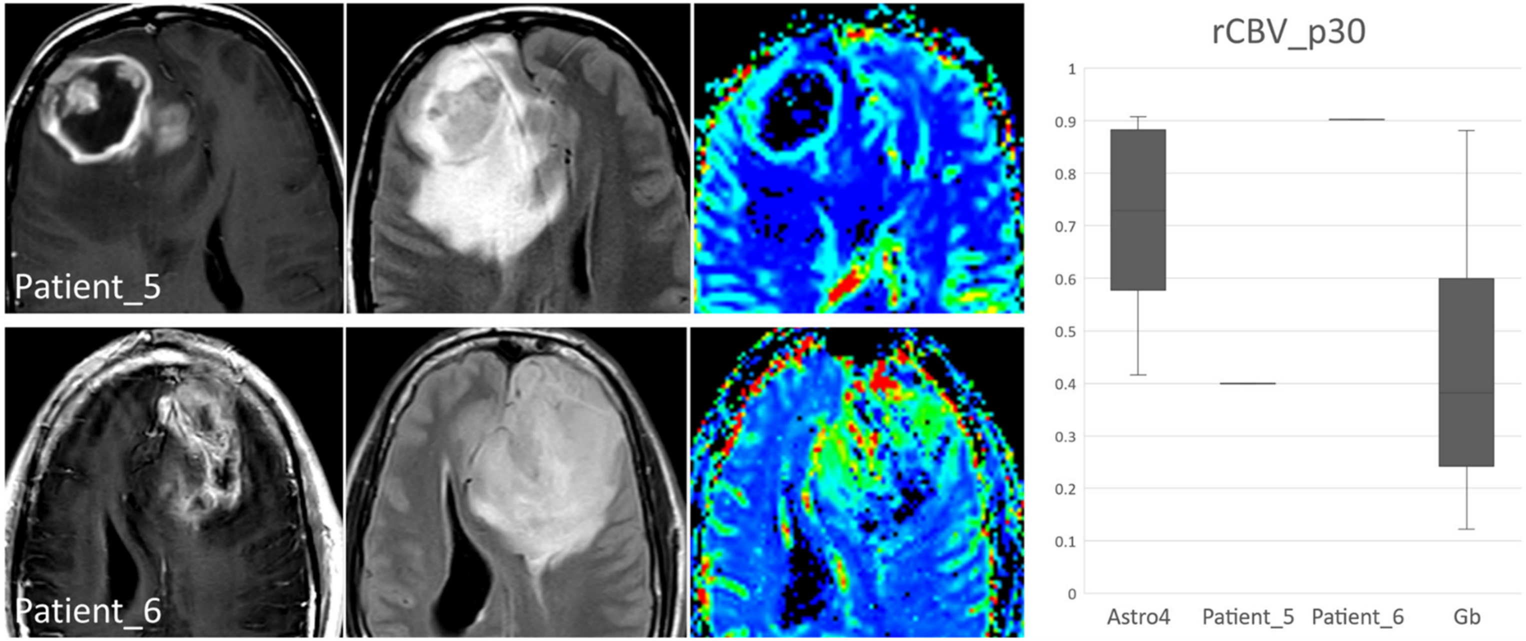
Patient_5
Patient_6
rCBV_p30
1
0.9
0.8
0.7
0.6
0.5
0.4
0.3
0.2
0.1
0
Astro4
Patient_5
Patient_6
Gb

Full range of rCBV p30 values for all the dataset.

**TYPE**	Astro4	Astro4	Astro4	Astro4	Astro4	Astro4	Astro4	Astro4	Astro4	Astro4
**rCBV p30**	0.730	0.902	0.710	0.577	0.908	0.611	0.416	0.729	0.882	0.428
**TYPE**	Astro4	Gb	Gb	Gb	Gb	Gb	Gb	Gb	Gb	Gb
**rCBV p30**	0.866	0.399	0.680	0.583	0.271	0.234	0.779	1.427	0.183	0.520
**TYPE**	Gb	Gb	Gb	Gb	Gb	Gb	Gb	Gb	Gb	Gb
**rCBV p30**	0.372	0.767	0.486	0.467	0.809	0.248	0.601	0.209	0.162	0.497
**TYPE**	Gb	Gb	Gb	Gb	Gb	Gb	Gb	Gb	Gb	Gb
**rCBV p30**	0.203	0.621	0.218	0.311	0.548	0.339	0.239	1.479	0.394	0.482
**TYPE**	Gb	Gb	Gb	Gb	Gb	Gb	Gb	Gb	Gb	Gb
**rCBV p30**	0.297	0.632	0.251	0.263	0.246	0.392	0.593	0.187	0.200	0.521
**TYPE**	Gb	Gb	Gb	Gb	Gb	Gb	Gb	Gb	Gb	
**rCBV p30**	0.881	0.848	0.121	0.296	0.128	0.850	0.217	0.259	0.266	
